# Hypermethylation of the G protein‐coupled receptor kinase 6 (GRK6) promoter inhibits binding of C/EBPα, and GRK6 knockdown promotes cell migration and invasion in lung adenocarcinoma cells

**DOI:** 10.1002/2211-5463.12606

**Published:** 2019-03-19

**Authors:** Sumei Yao, Dandan Wu, Jinliang Chen, Peng Wang, Xuedong Lv, Jianan Huang

**Affiliations:** ^1^ Department of Respiratory the First Affiliated Hospital of Soochow University Suzhou China; ^2^ Department of Respiratory the Second Affiliated Hospital of Nantong University China

**Keywords:** C/EBPα, DNA methylation, epigenetic regulation, GRK6, lung adenocarcinoma

## Abstract

We previously reported that the expression of G protein‐coupled receptor kinase 6 (GRK6) is significantly downregulated in lung adenocarcinoma (LADC) tissues, and low expression levels of GRK6 are correlated with poor survival prognosis. However, the specific regulatory mechanisms and functions of GRK6 in LADC remain unknown. Here, we report that GRK6 mRNA expression levels are downregulated in LADC tissues compared to those in matched adjacent non‐tumor tissues (*P* < 0.001). The promoter of the *GRK6* gene was found to be hypermethylated in LADC tissues, and its methylation was correlated with both GRK6 expression and pathology grade. *GRK6* promoter hypermethylation may predict shorter overall survival. Treatment with 5‐aza‐2′‐deoxycytidine significantly enhanced *GRK6* gene expression. Four binding sites of CCAAT/enhancer‐binding protein‐α (C/EBPα) in the CpG island of the *GRK6* gene promoter were predicted *in silico*, of which three sites were further confirmed by ChIP. Decreased binding of C/EBPα to binding sites 1, 3 and 4 of the *GRK6* gene promoter was observed in LADC tissues. Inhibition of C/EBPα significantly inhibited GRK6 expression, while overexpression of C/EBPα significantly promoted GRK6 expression. In addition, overexpression of *GRK6* significantly suppressed, while *GRK6* knockdown promoted cell migration and invasion. Overexpression of *GRK6* enhanced E‐cadherin expression and suppressed vimentin expression, and silencing of *GRK6* had the opposite effects. Furthermore, ectopic expression of *GRK6* significantly decreased matrix metalloproteinase (MMP) 2 and MMP7 protein expression levels. Our findings suggest that hypermethylation of the *GRK6* gene promoter suppressed binding of C/EBPα, thereby contributing to the promotion of cell migration and invasion. The methylation status of the *GRK6* promoter might be suitable for use as an epigenetic biomarker, and the C/EBPα–GRK6 signaling pathway may be a potential target for LADC.

**Abbreviations**
5‐Aza‐CdR: 5‐aza‐2′‐deoxycytidine
BSP: bisulfite sequencing PCR
C/EBPα: CCAAT/enhancer‐binding protein‐α
EMT: epithelial–mesenchymal transition
GRK6: G protein‐coupled receptor kinase 6
GRK: G protein‐coupled receptor kinase
LADC: lung adenocarcinoma
MMP: matrix metalloproteinase
MSP: methylation‐specific PCR
NSCLC: non‐small‐cell lung cancer
qPCR: quantitative PCR

Lung cancer is reported as the leading cause of tumor‐related deaths worldwide, with more than 1 million deaths reported annually, regardless of gender or ethnicity 1. Nearly 85% of all lung carcinomas are non‐small‐cell lung cancer (NSCLC) 2, 3, including lung adenocarcinoma (LADC), lung squamous cell carcinoma, lung large‐cell carcinoma and other, rare types. LADC is the most common histological subtype of NSCLC with increasing prevalence, followed by squamous cell carcinoma 4. Although NSCLC is one of the most extensively studied disorders of the past few years, it is a complex process that requires further investigation. Similar to other malignancies, the development and progression of NSCLC is a multistage process that involves both epigenetic and genetic changes 5. As one of the most common epigenetic modifications in mammalian genomes, DNA methylation plays critical roles in tumorigenesis 6. Alterations in DNA methylation status subsequently lead to the inactivation of gene expression. In particular, inactivation of tumor suppressor genes caused by hypermethylation of promoter regions has been shown to be important in the onset and progression of cancer 6, 7.

G protein‐coupled receptor kinases (GRKs) are a large protein kinase family that regulate the activity of G protein‐coupled receptors by phosphorylating their intracellular domains 8, 9. GRKs have been reported to participate in various human diseases, including homologous desensitization, opiate addiction, heart failure and malignant tumor development 8, 10, 11. G protein‐coupled receptor kinase 6 (GRK6), a GRK family member, has previously been shown to play a critical role in many diseases process 12, 13, 14. Thereafter, a new role of GRK6 in some cancer metastases was gradually revealed, such as medulloblastoma 15, multiple myeloma 14, and lung cancer 16. Our previous study implied that low GRK6 expression is found in LADC tissue and that GRK6 expression levels predict overall survival in LADC patients 17. However, the specific regulatory mechanisms and functions of GRK6 in LADC remain to be examined.

Our study aimed to investigate the epigenetic regulation mechanism of GRK6 expression and its biological functions in LADC. The results demonstrated that in LADC tissues the expression of GRK6 was low and that the *GRK6* gene promoter was hypermethylated and its methylation correlated with GRK6 expression and survival prognosis. Furthermore, the suppressed binding capability of CCAAT/enhancer‐binding protein‐α (C/EBPα) with a hypermethylated promoter of the *GRK6* gene led to the downregulation of GRK6 expression, which promoted cell migration and invasion. These observations provided the first evidence that the downregulation of GRK6 expression is mediated by the suppressed binding capability of C/EBPα with a hypermethylated promoter of the *GRK6* gene, thus contributing to the promotion of cell migration and invasion.

## Materials and methods

### Patients and tissue samples

Lung adenocarcinoma tissues were obtained from 54 patients who underwent resection surgery at the Second Affiliated Hospital of Nantong University and the Affiliated Hospital of Nantong University from 2009 to 2012. This study was approved by the Ethics Committee of the Second Affiliated Hospital of Nantong University and the Affiliated Hospital of Nantong University, and all procedures performed in this study were in accordance with the 1964 Declaration of Helsinki and its subsequent amendments. Written informed consent was obtained before tissue collection. None of the patients were treated with chemotherapy, radiotherapy or immunotherapy before surgery.

### RNA extraction and quantitative PCR

Total RNA was extracted with TRIzol reagent (Invitrogen/Life Technologies, Carlsbad, CA, USA) based on the manufacturer's instructions 18. The RNA (1 μg) was reverse transcribed using a PrimeScript RT reagent kit (Takara, Shiga, Japan). The conditions of reverse transcription were as follows: 1 cycle at 37 °C for 15 min and 1 cycle at 85 °C for 5 s. Then, the diluted cDNA (2 μL) was used for real‐time PCR using SYBR Green I (Takara) along with the primers, which were synthesized by Nanjing GenScript Co., Ltd (Nanjing, China). The conditions for the real‐time fluorescence quantitative PCR (qPCR) were as follows: 1 cycle at 95 °C for 5 min in the holding stage; 40 cycles at 95 °C for 15 s and 60 °C for 60 s in the cycling stage; and 1 cycle at 95 °C for 15 s, 60 °C for 1 min and 95 °C for 15 s in the melt curve stage. Thermal cycling and real‐time detection were conducted using StepOnePlus real‐time PCR systems (Applied Biosystems/Life Technologies). The expression level of each mRNA was calculated using the comparative ($2^{-\Delta \Delta C_{t}}$) method. The primer sequences were as follows: *GRK6* forward primer (5′‐AAAACACCTTCAGGCAATACCG‐3′) and reverse primer (5′‐AGGCCAAGCTCACTACAAACCTA‐3′) and *GAPDH* forward primer (5′‐CATGAGAAGTATGACAACAGCCT‐3′) and reverse primer (5′‐AGTCCTTCCACGATACCAAAGT‐3′).

### Methylation‐specific PCR

The CpG island methylation status of LADC tissue and cells was initially screened at *GRK6* gene promoter regions by methylation‐specific PCR (MSP). Extracted sample DNA was modified with bisulfite reagents according to the manufacturer's instructions (Zymo Research, Orange, CA, USA). This modification converted unmethylated cytosine to thymine, while methylated cytosine remained unchanged. A total of 20 ng of bisulfite‐modified DNA was subjected to PCR amplification and directly sequenced using an ABI3700 automated sequencing system (Applied Biosystems, Foster City, CA, USA). When the CpG sites in the region analyzed by MSP are methylated, the methylated (M) band can be observed. However, the unmethylated (U) band is present when the sites are unmethylated. Occasionally, both bands could be present if the sites are partially methylated. The MSP primers designed for the *GRK6* gene promoter were as follows: methylated forward primer (5′‐TGGGTGGAGAGAAATTATAATATTC‐3′) and reverse primer(5′‐TATAACCGTATTCCCTTAAATCGAC‐3′) and unmethylated forward primer (5′‐ GGTGGAGAGAAAT TATAATATTTGG‐3′) and reverse primer (5′‐ TATAA CCATATTCCCTTAAATCAAC‐3′).

### Bisulfite sequencing PCR

The status of methylation was further investigated by bisulfite sequencing PCR (BSP) 18. Bisulfite‐treated DNA was amplified by PCR using the following primers: GRK6‐BSP‐F (5′‐GTGGGTGGAGAGAAATTATAATATT‐3′) and GRK6‐BSP‐R (5′‐AATTTATTTAATCCACTTATATAACC‐3′). The PCR products were purified with a TIANgel Midi purification kit (TianGen Biotech, Beijing, China). Then, the PCR products were cloned into a pGEM‐T easy vector (Promega, Madison, WI, USA). Ten colonies were randomly chosen for plasmid extraction of DNA using a Promega Spin Mini kit (Promega) and were then sequenced by an ABI 3130 genetic analyzer (Applied Biosystems).

### Treatment with 5‐aza‐2′‐deoxycytidine

Lung cancer cell lines were inoculated in six‐well plates at a density of 5 × 10^6^ cells/well, cultured for 24 h, and then treated with 2 μm of the DNA methyltransferase inhibitor 5‐aza‐2′‐deoxycytidine (5‐Aza‐CdR; Sigma‐Aldrich, St Louis, MO, USA), which was added to the medium for three consecutive days.

### ChIP assay

The ChIP assay using a Chromatin Immunoprecipitation Kit (Sigma‐Aldrich) was based on the manufacturer's protocol 18. Briefly, the protein–DNA complexes were crosslinked with 1% formaldehyde, followed by nuclear fractionation and DNA shearing through sonication. Purified chromatin was immunoprecipitated using an anti‐C/EBPα antibody (1 : 500, ab40764; Abcam, Cambridge, MA, USA) or mouse immunoglobulin G (IgG, negative control). The antibody–protein–DNA complex was eluted from the beads, and the crosslinking was reversed by incubation after washing. The input fraction corresponded to 2% of the chromatin solution before immunoprecipitation. By bioinformatics analysis of the *GRK6* gene promoter, potential C/EBPα binding sites were identified, which are located −793/−784 bp, −514/−505 bp, −394/−385 bp and −198/−189 bp upstream of the transcription start site. Primers that were used to amplify the area containing these C/EBPα binding sites included the forward primer 5′‐CTGTTTCCTGCTGCGTGAAG‐3′ and the reverse primer 5′‐CCCTAGACCTCGGGATGAGT‐3′, resulting in a 183 bp fragment for the −793/−784 bp site. The forward primer5′‐CCGACCCAAGGGAATACG‐3′ and the reverse primer 5′‐TCTAGGGTTTTGTCGGGGAC‐3′ resulted in a 100 bp fragment for the −514/−505 bp site. The forward primer 5′‐GTCCCCGACAAAACCCTAGA‐3′ and the reverse primer 5′‐GGGACAATGGAGGTGGGTAC‐3′ resulted in a 169 bp fragment for the −394/−385 bp site. The forward primer 5′‐TACCCACCTCCATTGTCCC‐3′ and the reverse primer 5′‐GTCACCTCTTCCTGGTTGGTC‐3′ resulted in a 123 bp fragment for the −198/−189 bp site. The PCR conditions were as follows: 95 °C for 5 min, 37 cycles (95 °C for 30 s, 53–60 °C for 30 s, and 72 °C for 30 s), and 72 °C for 10 min. The products were observed using agarose gel electrophoresis. The signal intensity of the bands was calculated on grayscale images using imagej (National Institutes of Health, Bethesda, MD, USA).

### Cell culture and transfection

A549, A427 and H1299 LADC cell lines were obtained from the Cell Bank of Chinese Academy of Sciences (Shanghai, China) and cultivated in standard R10 medium (RPMI1640; Invitrogen, Karlsruhe, Germany) at 37 °C in a humidified atmosphere with 5% CO_2_ in air. The medium was supplemented with 10% FBS (v/v), 50 U·mL^−1^ penicillin, and 50 μg·mL^−1^ streptomycin, which were all obtained from Invitrogen (Germany). The cells were transfected using Lipofectamine 3000 (Invitrogen, USA) according to the manufacturer's instructions.

GRK6 or C/EBPα cDNA was cloned into the mammalian expression vector pcDNA3.1 (Invitrogen, USA). To inhibit the expression of GRK6, siRNA targeting GRK6 was synthesized by GeneChem (Shanghai, China). The target sequence of the GRK6 siRNA sequence (5′‐GCCGACUACCUCGACAGCAUCUACU‐3′) was used, and a scrambled sequence (5′‐TTCTCCGAACGTGTCACGT‐3′) was used as a negative control. The siRNA targeting C/EBPα and a non‐targeting control siRNA were purchased from RiboBio Co., Ltd (Guangzhou, China).

### Luciferase reporter assay

The *GRK6* promoter regions (the upstream 2000 bp of the transcriptional starting site) were constructed by amplifying genomic DNA and cloning the DNA into the pGL3‐basic vector. The mutated *GRK6* promoter reporter plasmids were constructed in the same manner. The assays were conducted using Lipofectamine^®^ 3000 (Invitrogen, USA) by transfecting the mutated *GRK6* promoter reporter plasmids, together with the basic pRL vector, into 16HBE cells in triplicate.

### Western blot analysis

Total proteins were extracted using RIPA lysis buffer supplemented with protease inhibitor (Solarbio, Beijing, China). Then, the extracts were separated by 10–12% SDS/PAGE and transferred to PVDF membranes. The membranes were blocked with 10% non‐fat milk powder in TBS for 2 h at room temperature and incubated with anti‐GRK6 (1 : 500, sc‐566; Santa Cruz Biotechnology, Dallas, TX, USA), anti‐C/EBPα (1 : 500, ab40764; Abcam), anti‐E‐cadherin (1 : 1000, ab1416; Abcam), anti‐vimentin (ab92547; Abcam), and anti‐β‐actin (1 : 5000, ab8226; Abcam) primary antibodies at 4 °C overnight. After three washes with TBST, the membranes were incubated with horseradish peroxidase‐conjugated goat anti‐mouse IgG (1 : 5000, sc‐2005; Santa Cruz Biotechnology) and horseradish peroxidase‐conjugated goat anti‐rabbit IgG (1 : 5000, sc‐20045; Santa Cruz Biotechnology). The signal intensity of the bands was calculated on grayscale images using imagej.

### Invasion and migration assays

We observed cell invasion and migration using a Transwell chamber assay (Corning Inc., Corning, NY, USA) either with or without a Matrigel (BD Biosciences, San Jose, CA, USA) coating. A total of 600 μL of culture medium with 10% FBS was added to the lower chamber, while the upper chambers were filled with serum‐free DMEM. After incubation for 48 h, cells invading the lower chamber were stained, fixed, photographed, and quantified by counting them at a magnification of ×200 from five different fields with each filter. Each experiment was repeated three times.

### Statistical analysis

Data analysis was conducted using spss software (SPSS Statistics version 17.0; SPSS Inc., Chicago, IL, USA). All data are reported as the mean ± SD of at least three independent experiments. Comparison between two groups was analyzed by Student's *t* test. A χ^2^ test was performed to determine the relationship between *GRK6* promoter methylation and clinicopathological features. Survival analysis was calculated by the Kaplan–Meier method. *P*‐values < 0.05 were considered statistically significant.

## Results

### GRK6 expression was significantly downregulated in LADC tissues

**Figure 1:**
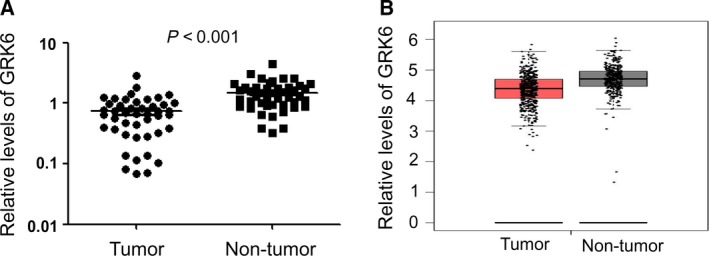
GRK6 expression is significantly downregulated in LADC tissues. (A) qPCR results showed that the expression of GRK6 was significantly decreased in LADC tissues compared with that in the adjacent non‐tumor tissues (*P *<* *0.001, paired Student's *t* test). (B) TCGA database analysis results showed a decrease in GRK6 expression in LADC tissues compared with that in non‐tumor tissues. The data represent the results from three independent experiments.

To determine the expression of GRK6 in LADC tissues, a qPCR assay was performed in 54 pairs of LADC tissues and matched adjacent non‐tumor tissues. Compared with adjacent non‐tumor tissues, the expression of GRK6 was downregulated in LADC tissues (*P *<* *0.001, Fig. 1A). Next, we found a decrease trend in GRK6 expression levels in LADC tissues compared with those in non‐tumor tissues using The Cancer Genome Atlas (TCGA) database analysis (Fig. 1B). These results imply that aberrant GRK6 expression might play an important role in the progression of LADC.

### Downregulation of GRK6 due to DNA hypermethylation in the promoter

**Figure 2:**
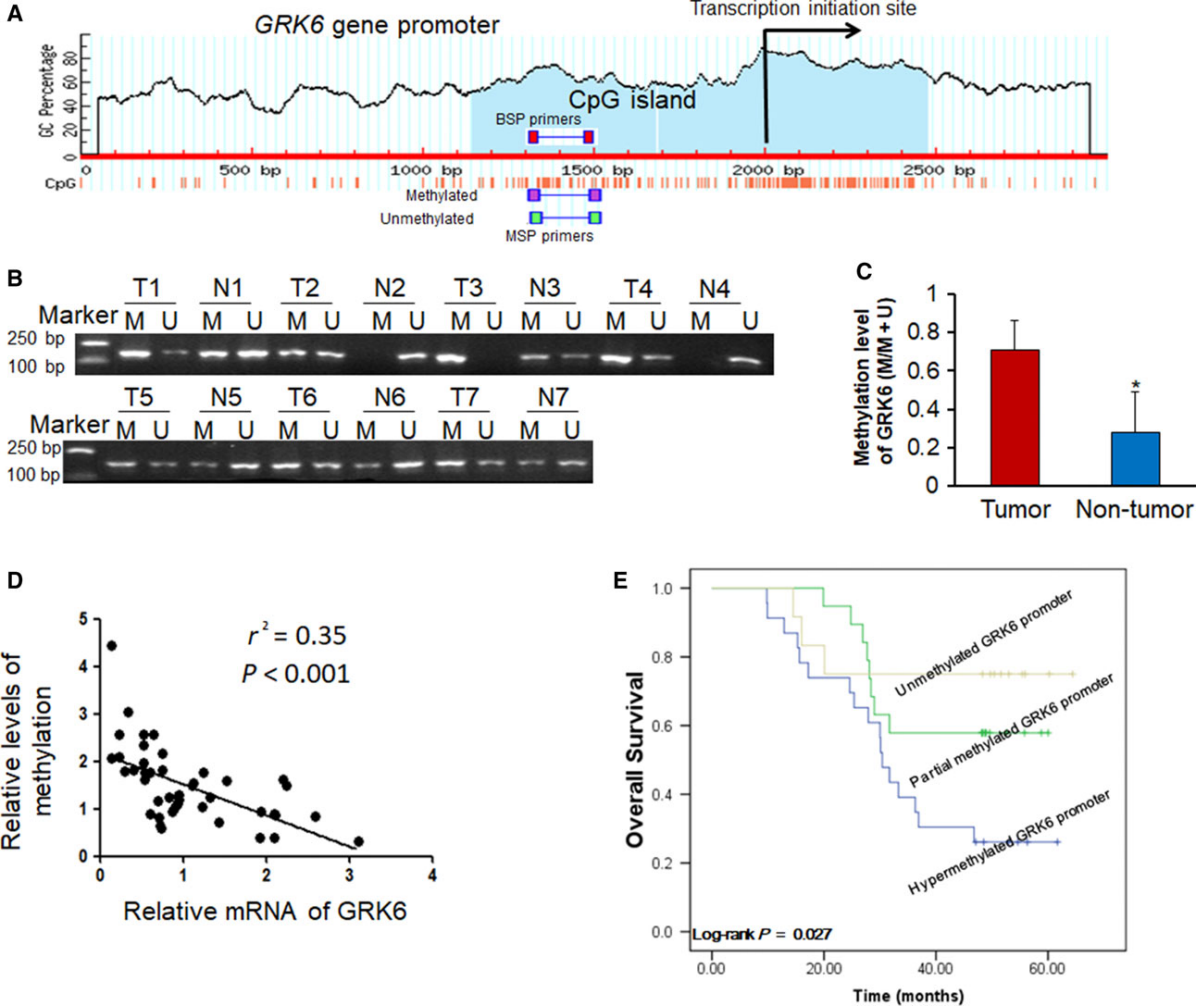
Downregulation of GRK6 expression in LADC tissues is associated with hypermethylation of the CpG island in the promoter. (A) A schematic map of the *GRK6* promoter region indicates the location of the CpG island (blue region). The positions for MSP and BSP primers are also indicated. (B) Representative results of methylation analysis by MSP in LADC and matched adjacent non‐tumor tissues. Case numbers are shown at the top. M, methylated primers; U, unmethylated primers. (C) The methylation level of the *GRK6* gene promoter in LADC tissues (Tumor) was higher than that in the matched adjacent non‐tumor tissues (Non‐tumor) in LADC patients (*P <* *0.01, paired Student's *t* test). Data are presented as means ± SD. (D) Correlation analysis between the methylation level of the *GRK6* gene promoter and gene expression (*r* ^2^ = 0.35, *P *<* *0.001). (E) Kaplan–Meier survival analysis of LADC patients with unmethylated, partially methylated and hypermethylated *GRK6* promoters (*P *=* *0.027, log‐rank test). The data represent the results from three independent experiments.

To identify whether the reduced expression of GRK6 in LADC tissues was due to DNA hypermethylation, online software (www.urogene.org/) was used to search for CpG islands in the *GRK6* gene promoter. After analysis, a CpG island located in the vicinity of the transcription start site was identified (Fig. 2A). A MSP assay was performed to analyze the methylation status of the CpG islands of the *GRK6* gene promoter in the LADC tissues and the adjacent non‐tumor tissues of 54 randomly selected cases. Representative MSP results of seven cases are shown in Fig. 2B. When the CpG sites are methylated, the methylated (M) band is apparent, but when the CpG sites are unmethylated, the unmethylated (U) band is apparent. Occasionally, both bands could be present if the sites are partially methylated. A higher methylation level was found in the 54 LADC tissues than in the adjacent non‐tumor tissues (*P *<* *0.05, Fig. 2C). Next, we evaluated whether there was a correlation between the methylation level and gene expression. Through correlation analysis, it was found there was a negative relationship between the methylation level of the *GRK6* gene promoter and gene expression in the LADC tissues (Fig. 2D).

An MSP assay of 54 LADC tissues showed *GRK6* promoter methylation, including hypermethylation and partial methylation, in 42 out of 54 LADC patients and was associated with pathology grade (*P *=* *0.024), whereas no correlation was found between the *GRK6* promoter methylation level and age, gender, smoking status, staging, and lymph node metastasis (Table 1). The prognostic value of *GRK6* promoter methylation was evaluated by Kaplan–Meier survival analysis. As shown in Fig. 2E, we found statistical differences in overall survival time among three groups with different methylation levels (*P *=* *0.027), while there were no statistical differences in overall survival time between unmethylated and partial methylation groups (*P *=* *0.492), suggesting that *GRK6* promoter hypermethylation may serve as a predictor for poor prognosis in LADC. However, *GRK6* promoter partial methylation has limited predictive value in overall survival time.

**Table 1:** Clinicopathological characteristics and *GRK6* methylation in 54 LADC patients

Clinicopathological characteristics	Number (*n* = 54)	*GRK6* methylation			*P* a
		Hypermethylation (*n* = 23)	Partial methylation (*n* = 19)	Unmethylated (*n* = 12)	
Age (years)					
≤ 60	25	10	7	8	0.252
> 60	29	13	12	4	
Gender					
Male	27	10	8	9	0.145
Female	27	13	11	3	
Smoking status					
Smoker	22	8	10	4	0.422
Never smoked	32	15	9	8	
Staging					
I + II	40	18	14	8	0.758
III + IV	14	5	5	4	
Pathology grade					
Well	10	2	2	6	0.024*
Moderate	28	15	10	3	
Poor	16	6	7	3	
Lymph node metastasis					
No	30	13	11	6	0.904
Yes	24	10	8	6	

^a^Chi‐square test. *Statistically significant difference.

**Figure 3:**
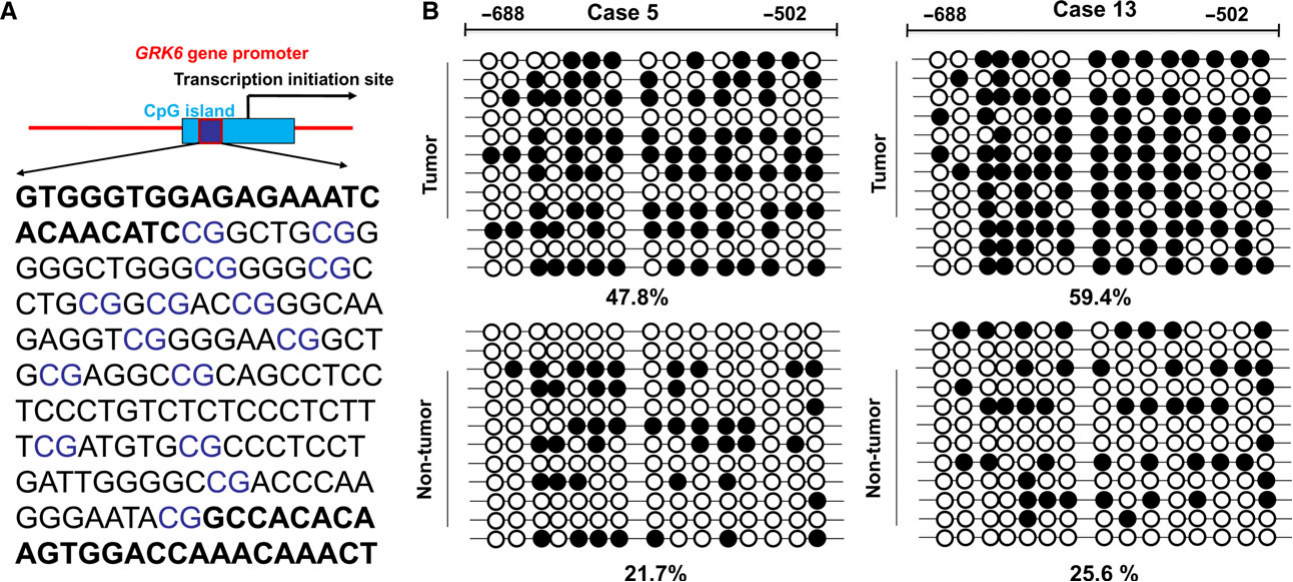
Analysis of the methylation status of the *GRK6* gene promoter in LADC tissues and adjacent non‐tumor tissues determined by a BSP assay. (A) A schematic map of CpG islands showing the locations of the 15 CpG sites in the *GRK6* gene promoter region. (B) Representative BSP results of methylation in LADC tissues and matched adjacent non‐tumor tissues of case 5 and case 13. Each row represents an individual cloned allele. Each circle represents a CpG site. The black circles show methylated CpG sites, and the white circles show unmethylated CpG sites. The data represent the results from three independent experiments.

To further evaluate the methylation level of CpG sites in the *GRK6* gene promoter, BSP was performed on the PCR products of the 187 bp fragment obtained after sodium bisulfite treatment. The 187 bp fragment contained 15 CpG sites (Fig. 3A; red labeling). Representative results of the BSP analysis for the *GRK6* gene promoter in LADC tissues and adjacent non‐tumor tissues are shown in Fig. 3B. The methylation frequencies of CpG sites within the *GRK6* gene promoter in LADC tissues and adjacent non‐tumor tissues were 52 ± 7.8% and 23 ± 6.5%, respectively. The methylation frequencies in the *GRK6* gene promoter in LADC tissues were significantly greater than those in the adjacent non‐tumor tissues. These results suggested that the reduced expression of GRK6 may be due to DNA hypermethylation of the CpG island in the promoter region.

### DNA methylation participates in GRK6 expression

**Figure 4:**
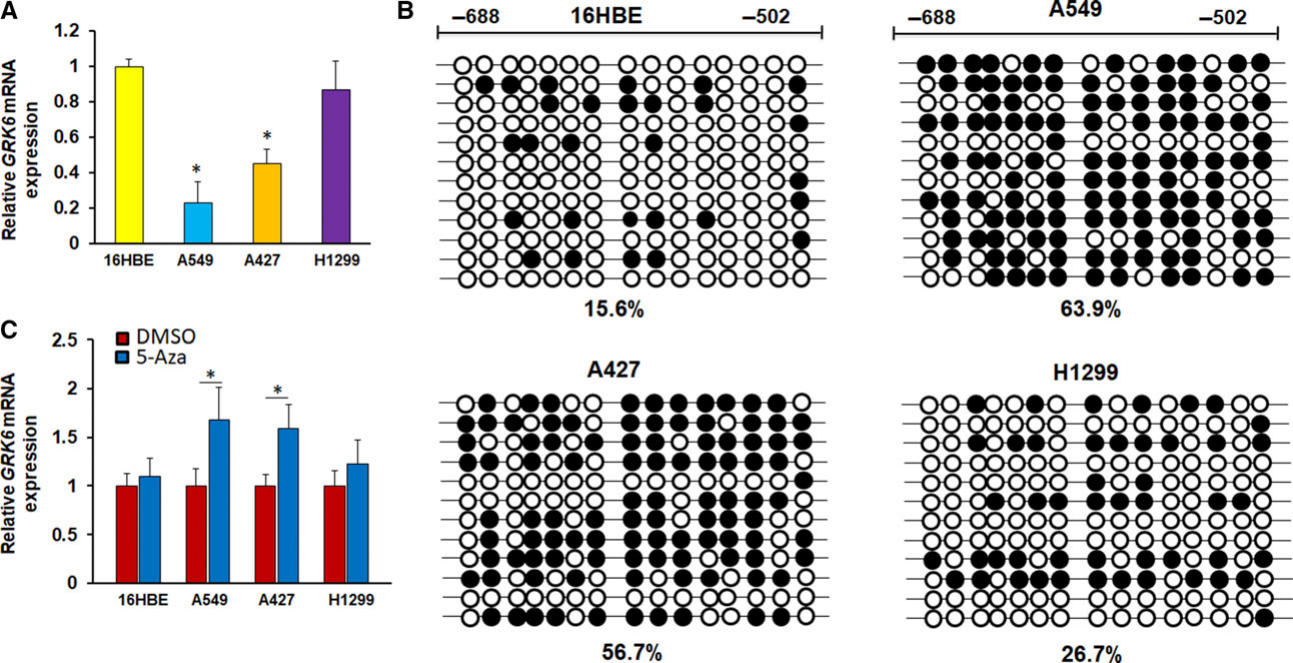
*GRK6* expression is induced by 5‐Aza‐CdR treatment in LADC cell lines. (A) qPCR results showed that the expression of the *GRK6* gene in A549 and A427 cells was significantly less than that in 16HBE cells (*P <* *0.05, Student's *t* test). Data are presented as means ± SD. (B) The methylation status of the *GRK6* gene promoter was analyzed by BSP in the different cells. (C) *GRK6* mRNA expression levels were analyzed by qPCR in the indicated cells treated with 5‐Aza‐CdR (*P <* *0.05, Student's *t* test). Data are presented as means ± SD. The data represent the results from three independent experiments.

\Quantitative PCR results showed that the expression of the *GRK6* gene in the A549 and A427 cell lines was markedly downregulated compared with that in the human normal bronchial cell line 16HBE (Fig. 4A). To identify whether the reduced GRK6 expression in the A549 and A427 cell lines was also due to DNA methylation, the DNA methylation of *GRK6* in lung cancer cell lines was also examined. The BSP detection region contained 15 CpG sites in the CpG island of the *GRK6* gene promoter region (Fig. 4B). The results showed that the promoter region of the *GRK6* gene had a higher degree of methylation in the cell lines A549 (63.9%) and A427 (56.7%) but a lower degree of methylation in the 16HBE cell line (15.6%). Furthermore, the DNA methyltransferase inhibitor 5‐Aza‐CdR was used to treat cells. The expression of GRK6 was upregulated in the A549 and A427 cells after treatment with 5‐Aza‐CdR, suggesting that DNA methylation may be involved in the regulation of GRK6 expression (Fig. 4C).

### Decreased binding of C/EBPα with the *GRK6* gene promoter

**Figure 5:**
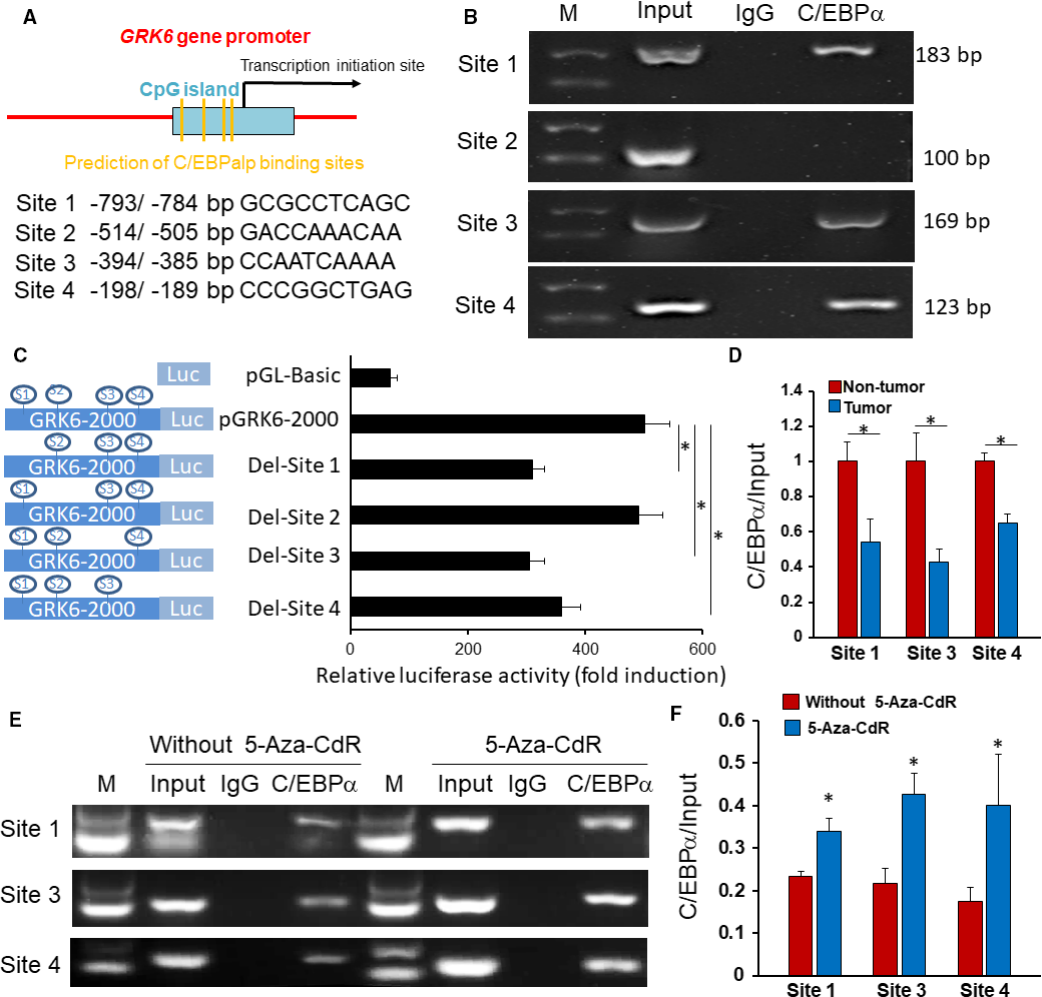
C/EBPα binding to the CpG island of the *GRK6* gene promoter. (A) This diagram shows the location of the predicted C/EBPα‐binding sites in the *GRK6* gene promoter. The sequences of the predicted binding sites for C/EBPα are listed. (B) A ChIP assay identified the direct binding of C/EBPα to the *GRK6* gene promoter. The ChIP‐enriched DNA fragments of the *GRK6* gene promoter using IgG and an anti‐C/EBPα antibody were amplified by PCR. Total input was used as a positive control. (C) Sequential deletion analysis identified C/EBPα binding sites in the *GRK6* gene promoter region and the relative luciferase activities were determined (*P <* *0.05, Student's *t* test). Data are presented as means ± SD. (D) The binding of C/EBPα with the *GRK6* gene promoter in LADC tissues was significantly enhanced compared with that in the corresponding non‐tumor tissues (*P <* *0.05, Student's *t* test). Data are presented as means ± SD. (E, F) The binding strength of C/EBPα to *GRK6* gene promoter increased significantly after 5‐Aza‐CdR treatment, compared with cells without 5‐Aza‐CdR treatment (*P <* *0.05, Student's *t* test). Data are presented as means ± SD. The data represent the results from three independent experiments.

DNA methylation is involved in gene expression regulation through the inhibition of related transcription factors binding with DNA 19. To investigate the related transcription factors that may participate in regulating GRK6 expression, the potential binding sites of related transcription factors in the CpG island of the promoter region were predicted using online prediction software (http://www.gene-regulation.com). We found that four potential C/EBPα binding sites (S1: −793/−784 bp; S2: −514/−505 bp; S3: −394/−385 bp; S4: −198/−189 bp; Fig. 5A) were located in the CpG island of the *GRK6* gene promoter. A ChIP assay was used to analyze the binding of DNA and C/EBPα. The results indicated that C/EBPα can bind to the CpG island of the *GRK6* gene promoter at S1, S3 and S4, whereas no binding was observed at S2 (Fig. 5B).

To examine the effect of these binding sites on GRK6 transcription, recombinant plasmids were constructed with wild‐type and mutated promoters of *GRK6*, and luciferase reporter gene assays were performed to investigate their effects. The normal pGRK6‐2000 luciferase promoter plasmid demonstrated enhanced transcription activity, and a significant reduction in transcription activity was found in groups with the deletion of S1, S3 or S4 in the promoter region (Fig. 5C). These results indicated that the S1, S3 and S4 C/EBPα binding sites are positive regulatory elements for *GRK6* transcription. Furthermore, we compared the binding of C/EBPα with the *GRK6* gene promoter between LADC tissues and adjacent non‐tumor tissues by using ChIP‐PCR. After C/EBPα antibody immunoprecipitation, the amount of DNA fragments amplified by different primer pairs (S1, primers for site 1; S3, primers for site 3; and S4, primers for site 4) significantly decreased in the LADC tissues compared with the adjacent non‐tumor tissues (Fig. 5D), indicating that the binding of C/EBPα with the *GRK6* gene promoter in the LADC tissues was specific and inhibited. Furthermore, the ChIP assay was repeated in lung cancer cell lines with or without 5‐Aza‐CdR, and we found that the binding strength of C/EBPα to the *GRK6* gene promoter increased significantly in the 5‐Aza‐CdR treatment group, compared with the untreated group (Fig. 5E,F).

### The effect of C/EBPα–/GRK6 signaling on cell migration and invasion

**Figure 6:**
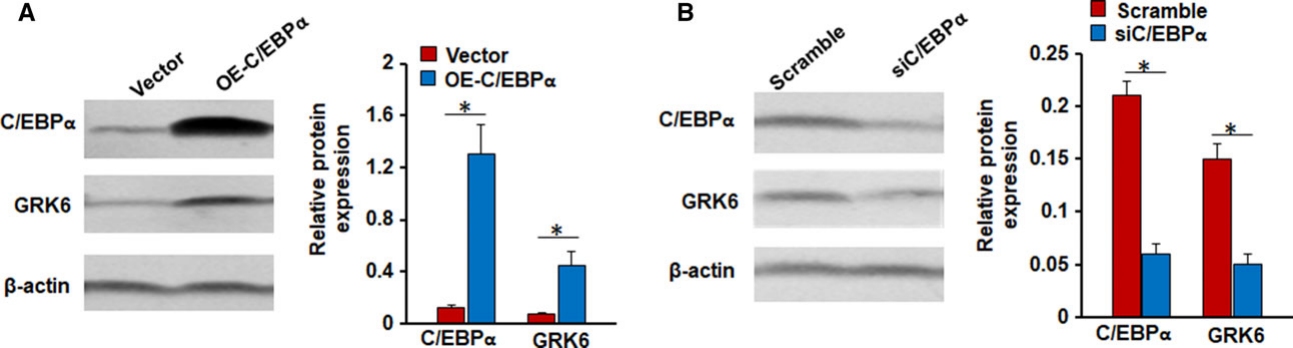
C/EBPα is involved in the regulation of GRK6 expression. (A) Western blotting analysis showed that the expression of C/EBPα in the cells transfected with pcDNA3.1‐C/EBPα significantly increased, and enhanced C/EBPα expression significantly upregulated the expression of the GRK6 protein. (*P < 0.05, Student's *t* test). Data were described as means ± SD. The data represent the results from three independent experiments. (B) The expression of C/EBPα in the cells treated with C/EBPα siRNA was significantly decreased compared with that of cells treated with scrambled siRNA, and inhibition of C/EBPα expression significantly downregulated the expression of the GRK6 protein. (*P < 0.05, Student's *t* test). Data were described as means ± SD.

To further confirm that C/EBPα is involved in the regulation of GRK6 expression, we silenced C/EBPα expression by RNAi and upregulated C/EBPα expression by pcDNA3.1‐C/EBPα. After transfection of siRNA or pcDNA3.1‐C/EBPα, western blotting analysis showed that the expression of C/EBPα in the cells treated with C/EBPα siRNA was significantly less than that of cells treated with scrambled siRNA. In contrast, the expression of C/EBPα in the cells transfected with pcDNA3.1‐C/EBPα was significantly increased compared with that of cells transfected with the pcDNA3.1 control vector. Furthermore, inhibition of C/EBPα expression significantly downregulated the expression of the GRK6 protein, while promoting C/EBPα expression significantly upregulated the expression of the GRK6 protein (Fig. 6).

**Figure 7:**
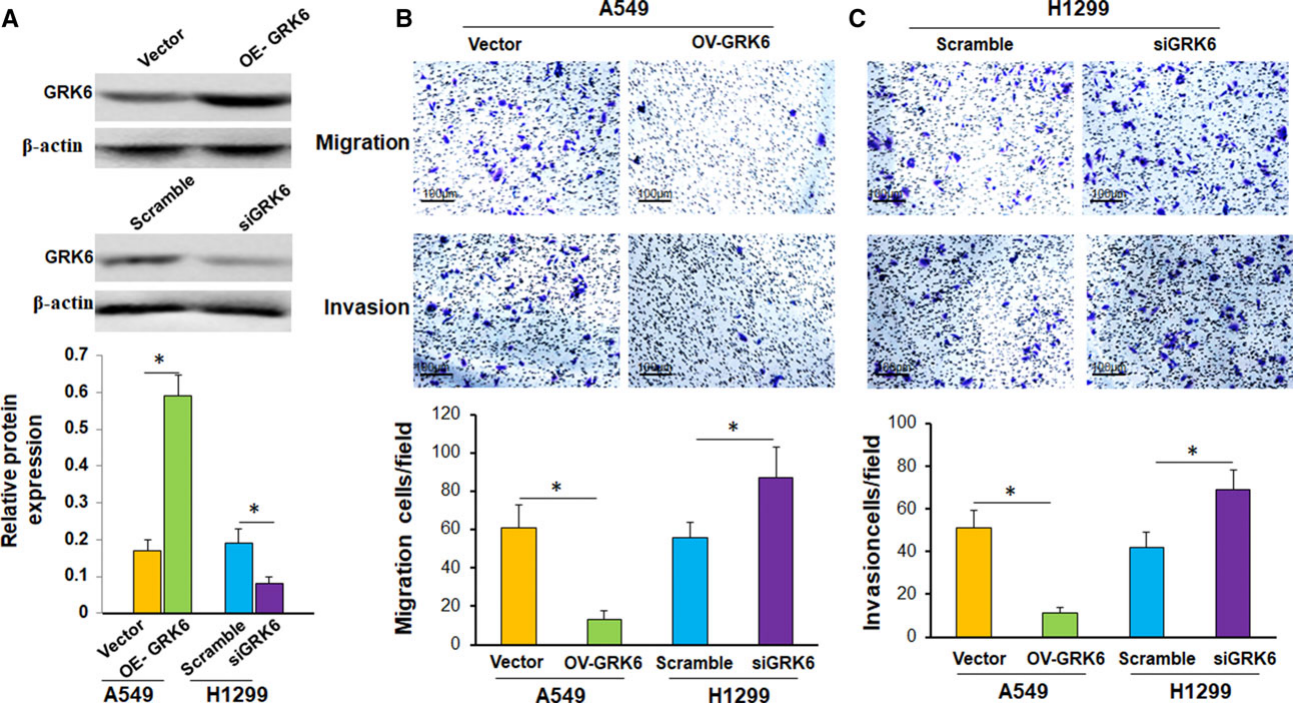
The effects of GRK6 on cell migration and invasion. (A) After transfection with siRNA or pcDNA3.1‐GRK6, the expression of the GRK6 protein decreased or increased significantly, respectively (*P <* *0.05, Student's *t* test). Data are presented as means ± SD. (B) Upregulation of GRK6 significantly inhibited cell migration and invasion (*P <* *0.05, Student's *t* test). Data are presented as means ± SD. (C) Downregulation of GRK6 significantly promoted cell migration and invasion (*P <* *0.05, Student's *t* test). Original magnification: ×200. Data are presented as means ± SD. The data represent the results from three independent experiments.

To further study the effects of GRK6 on cell migration and invasion, we also used siRNA and overexpression plasmids to inhibit and promote GRK6 expression. After transfection with siRNA, the expression of the GRK6 protein decreased significantly. In contrast, the expression of the GRK6 protein increased significantly after transfection with pcDNA3.1‐GRK6 (Fig. 7A). When the expression of the GRK6 protein was upregulated in the A549 cells transfected with pcDNA3.1‐GRK6, the migration assay results showed that the migratory ability was significantly suppressed, and the Matrigel invasion assay showed that the invasive ability was also significantly suppressed (Fig. 7B, *P <* *0.05). In contrast, when GRK6 expression was inhibited by siRNA in H1299 cells, the migratory and invasive abilities were significantly enhanced compared to those in the corresponding control group (Fig. 7C, *P < 0.05). These results implied that C/EBPα might regulate the expression of GRK6 and that GRK6 can regulate the migration and invasion abilities of LADC cells.

### GRK6 regulates epithelial–mesenchymal transition phenotypes and matrix metalloproteinase expression

**Figure 8:**
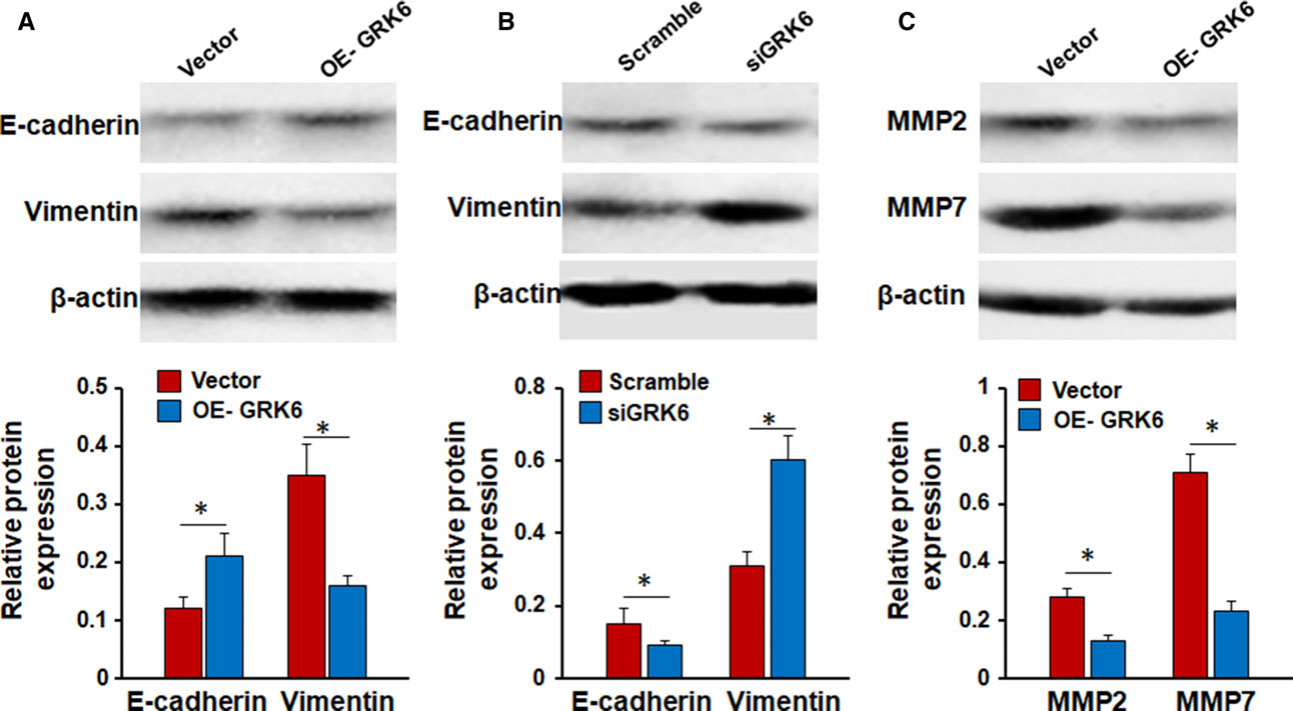
GRK6 affects EMT phenotypes and MMP expression. (A) GRK6 overexpression significantly increased E‐cadherin expression but inhibited the expression of vimentin (*P <* *0.05, Student's *t* test). Data are presented as means ± SD. (B) GRK6 knockdown reversed all of these changes (*P <* *0.05, Student's *t* test). Data are presented as means ± SD. (C) Upregulation of GRK6 significantly suppressed the expression of MMP2 and MMP7 proteins (*P <* *0.05, Student's *t* test). Data are presented as means ± SD. The data represent the results from three independent experiments.

Epithelial–mesenchymal transition (EMT) has been reported to promote the progression of tumors, mainly by inhibiting intercellular adhesion and increasing motility. E‐cadherin is a well‐known biomarker of epithelial cells, and vimentin is a biomarker of mesenchymal cells. To investigate the mechanism by which GRK6 promotes tumor cell migration and invasion, the expression of epithelial and mesenchymal biomarkers was examined after regulating GRK6 expression. The overexpression of GRK6 resulted in the upregulation of E‐cadherin expression and the downregulation of vimentin expression (Fig. 8A, *P < 0.05). In contrast, E‐cadherin expression decreased, and vimentin expression increased in GRK6‐downregulated cells (Fig. 8B, *P < 0.05). Matrix metalloproteinases (MMPs) belong to a family of extracellular proteinases that modulate various cellular processes, including cell migration and invasion and degradation of the extracellular matrix, in the process of tumor development and metastasis. MMP2 and MMP7, which are two typical MMP members, play critical roles in cell invasion and migration. Therefore, we tested the expression levels of MMP2 and MMP7 after modulating GRK6 expression and found that ectopic expression of GRK6 significantly inhibited the protein expression of MMP2 and MMP7 (Fig. 8C, *P < 0.05). These results suggest that GRK6 may participate in cell migration and invasion by regulating EMT and MMPs.

## Discussion

Lung adenocarcinoma is considered the most common and aggressive tumor with a varied prognosis. The biological mechanisms of LADC progression remain unclear. Therefore, the identification of genes involved in tumor cell proliferation, migration and invasion is exceedingly important. GRKs are a multifunctional kinase family and play a crucial role in homologous desensitization of G protein‐coupled receptors 20. GRK6 is the most recently identified member of the GRK family 21. Mounting evidence has suggested that GRK6 is also associated with malignancy progression 15, 22, 23, 24, 25. In our previous study, we showed that GRK6 expression was significantly downregulated in LADC and that the GRK6 expression level had an independent predictive value for overall survival. However, the regulation and role of GRK6 in LADC have been unknown until now.

GRK6 has distinct roles in various tumors. Its deficiency has also been observed in medulloblastoma tissues, which led to enhanced tumor progression depending on C‐X‐C chemokine receptor 4 signaling, while GRK6 upregulation diminished C‐X‐C motif chemokine 12‐induced migration 15. GRK6 has been identified as having a promoting role in cell adhesion and migration in prostate, breast and cervical epithelial cells 26. Similarly, GRK6 has been found to be overexpressed in hepatocellular carcinoma and was linked to poor prognosis 25. However, in another lung cancer study, inhibition of GRK6 was shown to promote the activity of host C‐X‐C chemokine receptor 2 signaling, followed by increased polymorphonuclear neutrophils infiltration and MMP release in the tumor microenvironment, facilitating angiogenesis and metastasis of lung cancer. In this study, the role of GRK6 in lung cancer cells showed that the loss of GRK6 expression may participate in cell migration and invasion by regulating EMT and MMPs. Therefore, we speculated that the contrasting function of GRK6 in specific cancers depends on specific receptor proteins or downstream pathways.

Although GRK6 has attracted increased attention for its role in tumorigenesis, there are few reports that have focused on the regulation of GRK6 in the biological processes of various cancers. Recent studies have shown that aberrant DNA methylation is a common regulatory mechanism that is attributed to decreased expression of tumor suppressor genes in oncogenesis. Therefore, we speculated that the methylation of CpG islands in the promoter region of *GRK6* may be a major mechanism of the promotion of LADC by GRK6. Our study suggested that the reduced expression of *GRK6* may be due to DNA hypermethylation of its CpG island in the promoter region. Treatment with 5‐Aza‐CdR significantly reversed *GRK6* gene expression. Therefore, we speculate that treatment with 5‐Aza‐CdR could be an effective treatment for LADC in the near future.

C/EBPα is a basic leucine zipper transcription factor involved in human cell proliferation and differentiation 27. Recent studies have found that it exhibits tumor‐suppressing characteristics in some cancers 28. In particular, a recent study focusing on lung cancer showed that C/EBPα impaired tumor growth of LADCs with low C/EBPα expression by targeting B lymphoma Mo‐MLV insertion region 1 homolog inhibition 29. Another study also demonstrated that C/EBPα, acting as a tumor suppressor, suppressed tumor cell invasion and migration in LADC cells by inhibiting β‐catenin‐mediated EMT *in vitro* 30. DNA methylation regulates target gene expression through the inhibition of related transcription factor binding with DNA 19. In this study, we found that C/EBPα can bind to the CpG island of the *GRK6* gene promoter at S1, S3 and S4. Our study demonstrated that C/EBPα, a transcription factor of *GRK6*, inhibited tumor cell migration and invasion in LADC cells by binding to the *GRK6* gene promoter. Moreover, the binding of C/EBPα with the *GRK6* gene promoter in LADC cells was inhibited, which may have been due to *GRK6* gene promoter hypermethylation that obstructed binding.

To investigate the effect of the loss of GRK6 expression on cell migration and invasion, the changes in expression of epithelial and mesenchymal biomarkers were analyzed after modulating the GRK6 expression level. Previously, Huan *et al*. 31 reported that C/EBPα suppresses hepatocellular cancer metastasis by downregulating the epidermal growth factor receptor/β‐catenin signaling pathway, which mediates EMT. Another recent study conducted by Lu *et al*. 30 suggested that C/EBPα inhibits cell migration and invasion by suppressing EMT in LADC cells. In our study, C/EBPα regulated EMT and MMPs through transcription regulation of *GRK6*, which is a novel mechanism of C/EBPα as a tumor suppressor in LADC. Therefore, a therapeutic approach targeting C/EBPα could be a promising therapy for LADC. However, the functional mechanism of C/EBPα in LADC development is still not fully understood and its investigation is of interest.

In conclusion, we have demonstrated that downregulation of GRK6 expression was modulated by the suppressed binding capability of C/EBPα with a hypermethylated promoter of the *GRK6* gene, which contributed to the promotion of cell migration and invasion. The methylation status of the *GRK6* promoter could be used as an epigenetic biomarker, and a demethylation treatment of the *GRK6* promoter region might be a potential therapy for LADC.

## Conflict of interest

The authors declare no conflict of interest.

## Author contributions

We declare that all authors have contributed significantly and all authors are in agreement with the content of the manuscript. Conception and design: JH; methodology: SY, DW, JC, PW; validation of data: SY, DW; analysis and interpretation of data: SY, DW; experimental studies, administrative, technical support (i.e. experimental work or editing data presentation): SY, DW, JC, PW; writing, review of the manuscript: SY, DW, JC, PW, XL, JH; supervision and project administration: JH. All authors read and approved the final manuscript.
